# Collision Localization and Classification on the End-Effector of a Cable-Driven Manipulator Applied to EV Auto-Charging Based on DCNN–SVM

**DOI:** 10.3390/s22093439

**Published:** 2022-04-30

**Authors:** Haoyu Lin, Pengkun Quan, Zhuo Liang, Ya’nan Lou, Dongbo Wei, Shichun Di

**Affiliations:** School of Mechatronics Engineering, Harbin Institute of Technology, Harbin 150001, China; linhaoyu@hit.edu.cn (H.L.); quanpengkun@hit.edu.cn (P.Q.); liangzhuo@hit.edu.cn (Z.L.); louyn@stu.hit.edu.cn (Y.L.); weidb@hit.edu.cn (D.W.)

**Keywords:** physical vehicle–robot interaction, cable-driven manipulator, collision localization, collision classification, model-independent method, automatic feature extractor, compensator

## Abstract

With the increasing popularity of electric vehicles, cable-driven serial manipulators have been applied in auto-charging processes for electric vehicles. To ensure the safety of the physical vehicle–robot interaction in this scenario, this paper presents a model-independent collision localization and classification method for cable-driven serial manipulators. First, based on the dynamic characteristics of the manipulator, data sets of terminal collision are constructed. In contrast to utilizing signals based on torque sensors, our data sets comprise the vibration signals of a specific compensator. Then, the collected data sets are applied to construct and train our collision localization and classification model, which consists of a double-layer CNN and an SVM. Compared to previous works, the proposed method can extract features without manual intervention and can deal with collision when the contact surface is irregular. Furthermore, the proposed method is able to generate the location and classification of the collision at the same time. The simulated experiment results show the validity of the proposed collision localization and classification method, with promising prediction accuracy.

## 1. Introduction

A recent trend in service robot research has emerged from the motivation of using robots in human-centered environments to develop robot assistants for use in human daily life and to automate certain processes and tasks that may be inconvenient for us as humans to perform [1,2,3]. Today, electric vehicles (EVs) are becoming increasingly popular in our daily lives, which brings potentially new scenarios applying service robots. Amid numerous service scenarios for EVs, the inconvenient EV charging connection scenario, which depends on manual operation, prompts researchers to think about realizing an automatic charging service for EVs with robots [4,5,6], which can promote the entire automation of EVs in the last mile with auto valet parking (AVP) technology. Among the many types of robots that can be applied in the automatic charging realm for EVs, cable-driven serial manipulators are of great interest to researchers due to their lightweight structure, large reachable workspace and potentially low cost [6,7].

In the EV automatic charging scenario applying robot assistants, safety issues are of primary concern. Similar to the emphasis of collaborative robots on human–robot safety in physical human–robot interaction (pHRI) [8], vehicle–robot safety should be considered in physical vehicle–robot interaction (pVRI). In the automatic charging process, contact between vehicles and robots is inevitable. The intentional contacts desired by the charging tasks, such as connecting charging plugs and charging ports, are usually quite safe. However, due to existing errors of positioning sensors after long-term service, accidental collisions, which lead to damage to vehicles, may occur. In this context, distinguishing intentional contacts from accidental collisions and problems related to the reaction after collision should be paid great attention. Unfortunately, to date, there is very limited research on these aspects in pVRI.

In pHRI, thus far, numerous studies on collision problems have been carried out, which can inspire solutions in collision handling in pVRI. In [9], a collision event handling pipeline is proposed. Based on the pipeline, a typical collision event handling procedure can be divided into three strategies, namely pre-collision phases, the intermediate process and post-collision phases. The expansion of such strategies involves various subphases, including collision avoidance [10,11,12], collision detection [13,14], collision localization [15,16], collision identification [17,18], collision classification [19] and collision reaction [20]. In the automatic charging scenario, the vehicles are usually stationary. In general, estimating the location of the collision and identifying whether the collision is accidental or intentional can provide more effective information for subsequent robot response in this scenario. Considering the mentioned conditions, this paper focuses on the collision localization and classification (CLC) problems of the end effector of the cable-driven robot, on which a feasibility study has been carried out in the field of EV automatic charging [6].

Different approaches for the individual component of CLC have been presented in the literature. Existing localization strategies can be divided into two subclasses [21]: model-based and model-independent methods. In model-based methods, a reduced model with respect to the actual system is usually constructed as the observer, which is applied to monitor the state of the actual system. A joint velocity observer is a typical observer-based method [9]. The core idea of this method is to construct a virtual model to estimate the joint velocity dynamically. Additionally, the same scheme is regarded as a disturbance observer, which can monitor the unknown external joint torque. However, the inverse inertia matrix is introduced in the construction of the filter equation of the observer, which leads to dynamic coupling and nonlinearity. This characteristic of the filter will lead to a poor localization effect. To avoid the inverse of the robot inertia matrix, in [20,22], generalized momentum observers in the monitoring methods are introduced. This scheme eliminates the need for an estimate of joint accelerations, which will introduce noise in the final results, and can decouple the estimation results. In other words, the final filter equation of the observer is a stable, linear, decoupled, first-order estimation of the external collision joint torque. Thus, compared with the joint velocity observer, the generalized momentum observer is more sensitive and effective.

However, model-based methods, to some degree, can achieve the collision localization of the motor-direct-driven robot effectively, such as localizing a single contact on one link and localizing multiple contacts on different links [23]. Nevertheless, existing model errors and disturbances from robot joint actuators may affect the accuracy of these methods in practice, especially when the contact points are located closely on the same link. Furthermore, for cable-driven robots of which the actuators and joints are separated from each other by a large distance [6,24], using signals from actuators and joints may introduce more delay in the collision localization of the end of the robot. Meanwhile, due to the requirement of commanded torques in model-based methods, for robots with passive joints, they are generally ineffective.

In model-independent methods, sensitive skins can be used to achieve effective collision localization [25,26]. This method converts contact forces into electrical signals, which results in high sensitivity for collision localization. The drawback of this approach is that the skins are difficult to deploy globally, and in harsh environments, where frequent contacts are required, the service life of such skins will be reduced. To date, benefiting from the progress in machine learning (ML), localization algorithms based on a neural network (NN) [17,21], a support vector machine (SVM) [27] or a fuzzy system [28] have greatly promoted the development of model-independent methods. However, according to “no free lunch Theorems” [29], the effectiveness of methods based on machine learning may be influenced by specific tasks, application scenarios and platforms.

As another individual component of CLC, collision classification, which is a significant issue in pVRI, however, lacks sufficient attention in pHRI, compared to the other components. Nowadays, there are two kinds of solutions to this task in pHRI, i.e., ML-based [19] and observer-based methods [30]. These two methods focus on either distinguishing humans’ intentional contact from accidental collision or differentiating humans from other items, in which the variation in the external active force inputs of humans can be the criterion. However, this criterion is usually not suitable for pVRI, especially in auto-charging scenarios, where vehicles remain static. To the best of the authors’ knowledge, few relevant studies have been conducted in this context.

The rest of this paper is organized as follows: Section 2 summarizes the related work and clarifies the main contributions of this paper. Section 3 describes the accidental collision in the auto-charging process and the vibration modeling of the cable-driven manipulator in the case of collisions occurring on the end effectors. Section 4 introduces the overall scheme of the collision data collection and labeling. Section 5 presents the architecture of our proposed CLC method. In Section 6, the proposed method is validated in simulated experiments with a specific cable-driven manipulator, and the discussion of the results is also presented. Finally, Section 7 concludes the paper.

## 2. Related Work

Recently, more work has tended to formulate CLC as a classification problem for different kinds of time series [17,21]. In the present study, a machine learning method based on artificial feature extraction proved effective in dealing with some simple collision localization and classification problems where the analyzed signals have obvious differences, such as identifying which link of the manipulator the collision occurs on, and determining whether the collision is intentional or accidental in pHRI. However, when the collision locations to be identified are very close and the analyzed signals are highly similar, the above method may not be able to perform well. Since deep learning methods based on automatic feature extraction show promising results in addressing time-series classification problems, a large amount of related work can also provide inspiration for solving such CLC problems, such as physical examination using ECG [31,32,33], EEG pattern analysis [34,35] and the fault diagnosis of rolling bearings [36,37]. In these applications, the automatic extractor is usually composed of deep networks such as deep CNN or multilayer LSTM. As is well known, the depth of the network can influence the performance of the models to some degree. In general, the deeper the network, the better the classification ability. However, too many layers usually mean high training time costs.

With the development of transfer learning, using deep networks as extractors combined with traditional machine classifiers has been applied in many fields. In [38], a pre-trained GoogLeNet is applied as the extractor for brain MRI images, and the SVM and kNN are used as classifiers. The results show that the effect of the combination is promising compared with using pre-trained GoogLeNet alone. Similarly, in [39], a pre-trained VGG-16 combined with the SVM also achieves excellent results on the pulmonary nodule classification task. Applying a pre-trained model as a feature extractor can reduce the training time to some extent. However, these schemes do not change the high cost and high memory footprint characteristics of such models in application. For robots with a lower cost in general, such methods are not friendly enough. On the other hand, the input form required by the transfer models should correspond to their own structures. It is usually not flexible enough for cases where the input form required by the scenario and the input form required by the transferred model are inconsistent. Although the above methods have some limitations, it is indeed enlightening that a combined model can achieve better results in some tasks. Based on this idea, a model combining a simple convolutional neural network with the SVM is designed for CLC problems in the pVRI scenario, which can use raw data directly, without additional transformation, and is relatively computation-cost-effective. The main contributions of this paper are as follows:

1. Vibration signals of the elastic compensator were first introduced to realize CLC for the end effector of the cable-driven manipulator in the EV auto-charging process.
2. Unlike other works using torque sensors at the end of the manipulator, our data were collected using an IMU installed on the end effectors. In more detail, the end effector of the platform that we used for data collection was connected to the manipulator using an elastic compensator, and thus, the collected signals mainly contained the vibration information coming from the compensator when collision occurred.
3. A quantitative description method was first proposed for the contact position in the collision in the EV auto-charging scenario, which is called the collision point. By using this description method, the contact position can be clearly described when the contact surface is irregular, which is useful for collision data labeling.
4. An automatic feature extraction method combining a simple CNN with the SVM was proposed to realize collision classification and localization at the same time, which is able to isolate the influence of joint configurations on the prediction. Considering the input of the model, there is no need to covert time series into images, and raw data can be directly used.

## 3. Accidental Collision Illustration and Vibration Modeling

This section addresses accidental collisions during charging processes and the vibration presentation of the manipulator after contact occurrence. The equipment that we are concerned with here is a planar 3-DOF cable-driven manipulator with a slide platform at the bottom [6]. To satisfy the requirement of charging, an elastic compensator, which was used to connect the charger to the manipulator, was installed at the end of the manipulator.

### 3.1. Accidental Collision Illustration

**Figure 1:**
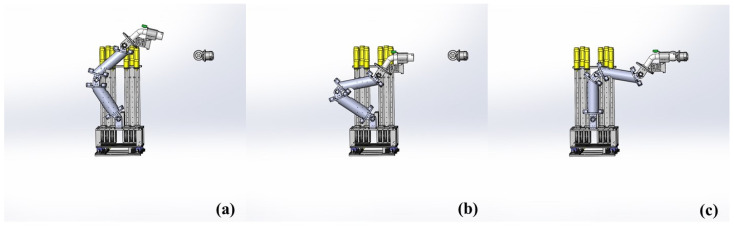
(**a**) The process of charging port localization; (**b**) the pre-insertion stage; (**c**) the process of inserting the charger into the charging port or plugging out the charger.

As shown in Figure 1, the whole charging process can be divided into four stages:

1. Charging port localization;
2. Movement of the charger to the pre-insertion place;
3. Inserting the charger into the charging port;
4. Pulling out the charger, which is an inverse process relative to Stage 3.

Due to the fact that abnormal insertion processes may cause fatal destruction, among these four stages, we focus far more on the second and the third stages. According to our insertion strategy, in the second stage, the end effector of the manipulator moves close to the pre-insertion place, which is calculated using the localization system in the first stage. Generally, in the stable state of the second stage, the charger usually maintains a certain distance from the charging port. In the third stage, the charger will be inserted into the charging port in a straight line at a constant speed. However, in these two processes, the precision of localization system calibration, the positioning accuracy of the localization system and the tension of the cables in the motion may seriously affect the final insertion accuracy. The factors mentioned above usually worsen after long-term service, and then accidental collisions will occur.

There are multiple possible scenarios of accidental collisions on different parts of vehicles during charging processes. In this paper, we only focus on the collisions occurring around the charging port due to a lack of insertion precision. These accidental collisions can be broadly divided into two cases:

1. The first case is that, when the central axis of the charger and the central axis of the charging port are not collinear, after the contact between the charger and the charging port, the charger can still slide into the charging port, and the deflection angle of the elastic compensator is small, which will not cause plastic deformation of the elastic compensator;
2. The second case is that, when the deviation between the central axis of the charger and the central axis of the charging port is too large, after the contact between the charger and the charging port contact, the charger cannot be inserted into the charging port, or even if it can be inserted, plastic deformation occurs easily in the elastic compensator, which will cause permanent damage to the end effector.

In practice, Case 1 is usually acceptable, while Case 2, which may cause permanent damage to the system, should be avoided. According to the above description, the standard to distinguish the above two cases is the deviation between the central axis of the charger and the central axis of the charging port. Different deviations may affect the degree of the damage of the end effector after collision, and, in general, the acceptable range of the deviation depends on the current industry standards [40] and the compensation capability of the compensator. Furthermore, due to the fact that the deviations affect our data collection scheme, the relative context will be illustrated in detail in Section 4.

### 3.2. Vibration Modeling of the Manipulator

**Figure 2:**
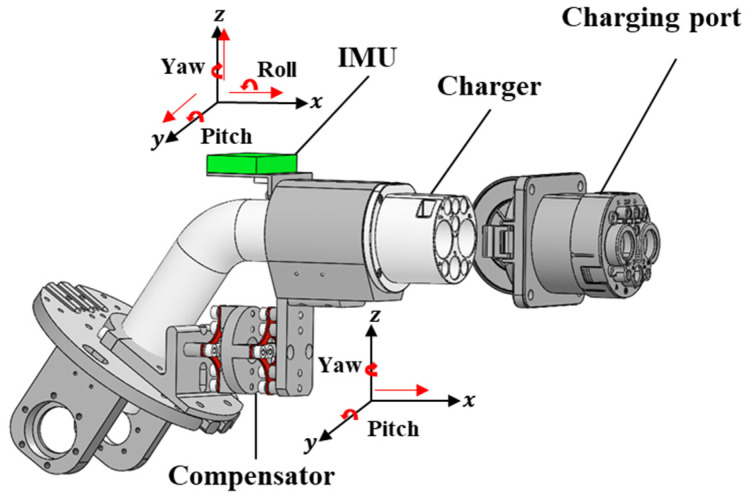
Vibration signal collection during insertion process.

In this paper, collision analysis was carried out based on the vibration signals of the compensator. As shown in Figure 2, an Inertial Measurement Unit (IMU) fixed on the top of the charger was used to collect the vibration signals coming from the compensator indirectly, which contained 3-axis acceleration and 3-axis angular velocity.

**Figure 3:**
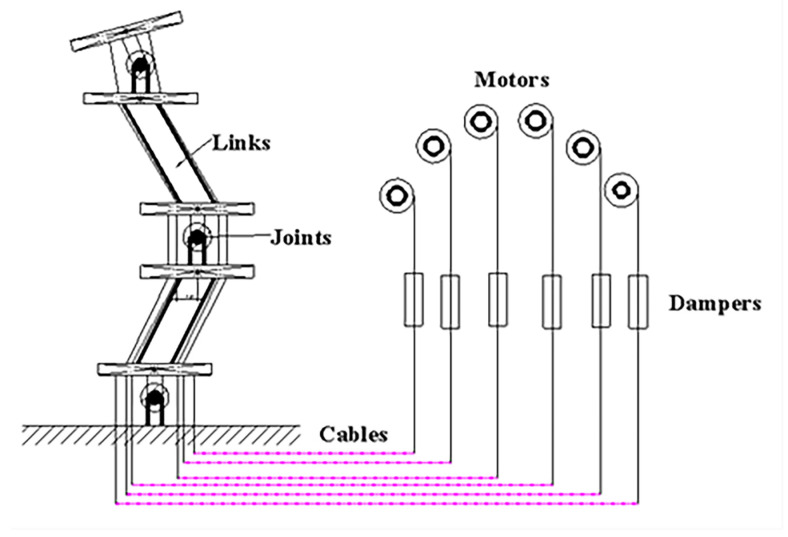
Illustration for the simplified planar 3-DOF cable-driven manipulator.

It is worth noting that the collected vibration signals also contain the vibration information from other parts of the manipulator, of which the influence should not be ignored. In order to consider the above influence on terminal collision signal collection, the vibration modeling of the manipulator without the end effector should be analyzed. During the insertion stage, the slide platform is fixed at a certain place. Thus, as shown in Figure 3, the model of the manipulator can be simplified as a planar 3-DOF cable-driven manipulator.

Elastodynamic analysis is an effective method for dealing with the vibration of the kinematic chain mechanism [21,41]. In this method, critical mechanical components can be simplified with stiffness, viscous and mass parameters. Thus, at the moment of a low-speed collision, the dominant vibration structures of the N-DOF cable-driven manipulator can be considered as elastic bodies with certain stiffness and viscous coefficients when the cables are in tension.

As in the case mentioned above, we consider a planar N-DOF manipulator with *n* dominant vibration structures composed of cables and joints. Its axis displacement vector can be defined as:

(1) $$q=\left[\begin{array}{c} q_{D}\\ q_{J} \end{array}\right]\in {\mathbb{R}}^{2N}$$

where $q_{D}={\left[q_{1},q_{2},\ldots ,q_{n}\right]}^{T}$ is the vibration deviation, and $q_{J}={\left[q_{n+1},q_{n+2},\ldots ,q_{2n}\right]}^{T}$ denotes the joint displacements. In a low-speed process, we assume that the equilibrium point of the deviation is $q_{J}$. The dynamic equation of the manipulator can be written as:

(2) $$M\left(q\right)\ddot{q}+C\left(q,\dot{q}\right)\dot{q}+G\left(q\right)=\left[\begin{array}{c} \tau_{D}\\ \tau_{C}+\tau_{f} \end{array}\right]$$

The variables $\tau_{D},\;\tau_{C}$ and $\tau_{f}$ denote the joint torque generated by structure deformation, cables and friction, respectively. Meanwhile, joint torque generated by structure deformation can be represented as follows:

(3) $$\tau_{D}=K_{p}\left(q_{J}-q_{D}\right)-K_{v}\dot{q_{D}}$$

where the $K_{p}$ and $K_{v}$ matrices denote the stiffness and viscous coefficients of the dominant vibration structures, respectively.

We assume that the feedback control loop can compensate for the gravity and friction, and the Coriolis and centrifugal effects caused by structure deformation can be ignored. Furthermore, in low-speed movement, $q_{D}$ is usually much smaller than $q_{J}$. Hence, the inertia matrix M(q) is mainly determined by $q_{J}$. Then, the detailed form of the dynamic function of the manipulator can be expressed as follows:

(4) $$\left[\begin{array}{c} \tau_{C}\\ \tau_{J} \end{array}\right]=\left[\begin{array}{cc} M\left(q_{J}\right) & 0\\ 0 & M\left(q_{J}\right) \end{array}\right]\left[\begin{array}{c} {\ddot{q}}_{D}\\ {\ddot{q}}_{J} \end{array}\right]+\left[\begin{array}{cc} 0 & C_{D}\left(q_{J},{\dot{q}}_{J}\right)\\ 0 & C_{J}\left(q_{J},{\dot{q}}_{J}\right) \end{array}\right]\left[\begin{array}{c} {\dot{q}}_{D}\\ {\dot{q}}_{J} \end{array}\right]+\left[\begin{array}{c} J_{D}^{T}\\ J_{J}^{T} \end{array}\right]F_{ext}$$

where $F_{ext}$, $J_{D}^{T}$ and $J_{J}^{T}$ denote the collision torque vector and the contact Jacobian matrix to dominant vibration structures and cables, respectively. Then, substituting (1) into (2), we have

(5) $$\left[\begin{array}{c} 0\\ \tau_{J} \end{array}\right]=\left[\begin{array}{cc} M\left(q_{J}\right) & 0\\ 0 & M\left(q_{J}\right) \end{array}\right]\left[\begin{array}{c} {\ddot{q}}_{D}\\ {\ddot{q}}_{J} \end{array}\right]+\left[\begin{array}{c} K_{p}\\ 0 \end{array}\right]\left(q_{J}-q_{D}\right)+\left[\begin{array}{cc} K_{v} & C_{D}\left(q_{J},{\dot{q}}_{J}\right)\\ 0 & C_{J}\left(q_{J},{\dot{q}}_{J}\right) \end{array}\right]\left[\begin{array}{c} {\dot{q}}_{D}\\ {\dot{q}}_{J} \end{array}\right]+\left[\begin{array}{c} J_{D}^{T}\\ J_{J}^{T} \end{array}\right]F_{ext}$$

Denoting $y_{1}=q_{D}-q_{J}$, $x_{1}=\left[\begin{array}{c} {\dot{q}}_{D}-{\dot{q}}_{J}\\ q_{D}-q_{J} \end{array}\right]$, and $y_{2}={\dot{q}}_{J}$, $x_{2}=\left[\begin{array}{c} {\dot{q}}_{J}\\ q_{J} \end{array}\right]$, we can then obtain the state–space equation as follows:

(6) $$\{\begin{array}{c} {\dot{x}}_{1}=A_{1}x_{1}+B_{11}y_{2}+B_{12}F_{ext}\\ {\dot{x}}_{2}=A_{2}x_{2}+B_{21}u+B_{22}F_{ext}\\ y_{1}=C_{1}x_{1}\\ y_{2}=C_{2}x_{2} \end{array}$$

where u is the active torque generated by cables. In a low-speed movement, when the system is subjected to external shocks, it is assumed that ${\dot{q}}_{D}\gg {\dot{q}}_{J}$ and ${\ddot{q}}_{D}\gg {\ddot{q}}_{J}$. Then, the related parameter matrix can be expressed as follows:

(7) $$\begin{array}{c} A_{1}=\left[\begin{array}{cc} -M{\left(q_{J}\right)}^{-1}K_{v} & -M{\left(q_{J}\right)}^{-1}K_{p}\\ I & 0 \end{array}\right],\\ A_{2}=\left[\begin{array}{cc} -M{\left(q_{J}\right)}^{-1}C_{J} & 0\\ I & 0 \end{array}\right],\\ B_{11}=\left[\begin{array}{c} -M{\left(q_{J}\right)}^{-1}C_{D}\\ 0 \end{array}\right],\\ B_{12}=\left[\begin{array}{c} -M{\left(q_{J}\right)}^{-1}J_{D}^{T}\\ 0 \end{array}\right],\\ B_{21}=\left[\begin{array}{c} -M{\left(q_{J}\right)}^{-1}\\ 0 \end{array}\right],\\ B_{22}=\left[\begin{array}{c} -M{\left(q_{J}\right)}^{-1}J_{J}^{T}\\ 0 \end{array}\right],\\ C_{1}={\left[\begin{array}{c} 0\\ I \end{array}\right]}^{T},\\ C_{2}={\left[\begin{array}{c} I\\ 0 \end{array}\right]}^{T} \end{array}$$

Then, the transfer function from $F_{ext}$ to $y_{1}$ can be expressed as follows:

(8) $$P\left(s\right)=\frac{Y\left(s\right)}{F\left(s\right)}=C_{1}{\left(sI-A_{1}\right)}^{-1}\left[B_{12}+B_{11}C_{2}{\left(sI-A_{2}\right)}^{-1}B_{22}\right]$$

Due to the fact that the inertial matrix is positive definite, we can deduce that $rank\left(A_{1}\right)=2n$ and $rank\left(A_{2}\right)=n$. Then, according to the conclusion of [21], the natural frequency of vibration along the manipulator is determined by the eigenvalues of $A_{1}$ and $A_{2}$. With regard to the cable-driven manipulator, $M\left(q_{J}\right)$, $K_{v}$ and $K_{p}$ are related to the joint displacement. This means that the natural frequency of the system will vary with the joint displacement. Thus, when collecting vibration signals, the joint configuration should be considered.

## 4. Data Collection and Labeling

In this section, we introduce the overall data collection and labeling scheme. To collect reasonable collision information, collision position description is necessary. When the charger and the charging port collide, the contact surface is usually irregular. Thus, it is difficult to describe collision positions and label collected vibration signals according to actual contact surfaces. To solve the problem above, we used the collision point, which is the intersection of the charger central axis and the front of the charging port, to represent the collision position. In our case, we focused on the situation in which the charger’s central axis is perpendicular to the front of the charging port before collision occurs.

### 4.1. Data Collection Scheme

**Figure 4:**
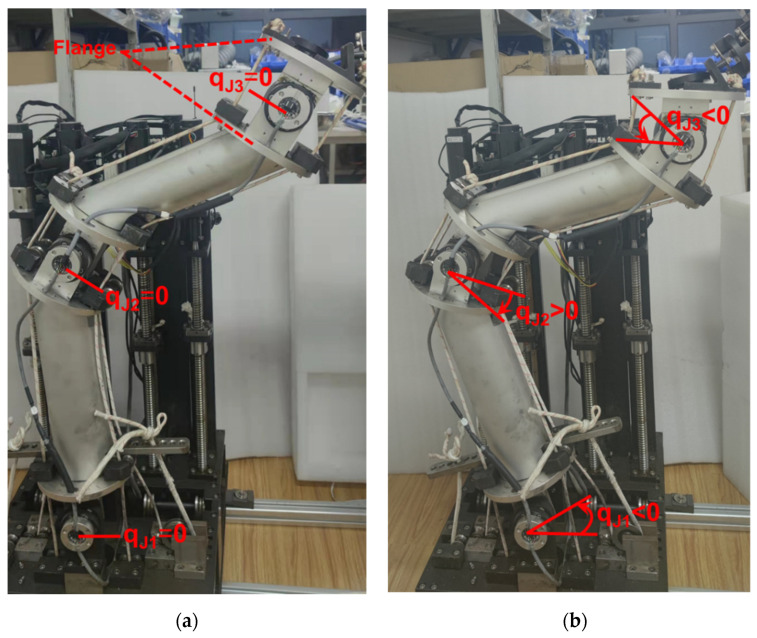
(**a**) The cable-driven manipulator at the state of zero joint angles; (**b**) the cable-driven manipulator at the state of non-zero joint angles.

In this paper, collision points were designed by introducing artificial systematic deviations into the center axis alignment status, in which the charger’s central axis and the charging port’s central axis were collinear. Considering the influence of the joint configuration at the moment of collision, we divided the collision points into four groups based on the differences between the joint configurations. Theoretically, the generalized joint configuration, among which the displacement of the slide platform is considered, maps the collision point in a one-to-one way. Thus, the joint configuration of the central axis alignment status can be regarded as the standard of group division. Meanwhile, in order to ensure the consistency of group division, we chose the joint configuration of the pre-insertion state, instead of that at the moment of collision, as the standard of group division. As shown in Figure 4a, in the experiment, the joint angle was zero when the two flanges of one joint were parallel. As shown in Figure 4b, the joint angle generated by clockwise rotation of the joint was defined as positive. According to the above definition, the joint configurations of the four groups are listed in Table 1.

**Figure 5:**
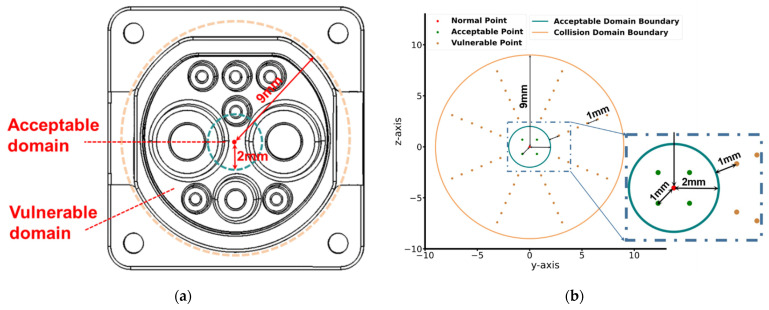
(**a**) The acceptable domain and the vulnerable domain of the charger; (**b**) the distribution of the designed collision points.

To construct a data set for the proposed CLC method, we needed to design a collision domain for the experiment. Here, the collision domain was set as a 9-mm-diameter circle, as shown in Figure 5a, of which the center was the center of the front of the charging port. According to Case 1 and Case 2 mentioned in Section 3, the collision domain can be divided into two subdomains: the acceptable domain and the vulnerable domain. The sizes of these two subdomains were both defined based on practical experience. In our design, the diameter of the acceptable domain was set to be 2 mm and the rest of the collision domain was the vulnerable domain, as shown in Figure 5a. In the whole collision domain, 53 collision points in each group were designed, as shown in Figure 5b. In the acceptable domain, 5 points were set, among which four green points were defined as *acceptable* points. The red center point here was defined as the *normal* point, referring to the situation in which the charger can be plugged into the charging port without any collisions before the charger comes into contact with the inner cavity of the charging port. To illustrate, center points in different groups were translated into the same coordinate system here. In the following, we distinguish them by group numbers. In the vulnerable domain, continuous contact after impact may cause great damage to the system. In this domain, 48 collision points were set, which were defined as *vulnerable* points. The above three cases could also be defined as *contact* against the situation called *free*, where no collisions occurred during the movement.

### 4.2. Segment and Labeling Scheme

In the experiment, consecutive collisions were conducted for one collision point in each group. One sample collected in this way contains information on multiple collisions. Thus, the signal in one sample should be split into different segments, among which each single segment maps one single collision for a collision point. We denote the length of the segments as l and the effective period as ep, with the unit of ms. Effective period indicates the time period that could be used to effectively analyze the collision. To be able to include the information of the transition from the free state to the impact in the effective period, the effective period should start slightly before the occurrence of contact, which is defined as the pre-collision period. Considering that there is continuous contact after an impact, the ending position of the effective period changes corresponding to the influence of the continuous contact on the validity of the analysis. In general, l determines how much signal information is included in segments, and it should be long enough so that a high-quality effective period can be extracted from segments. Here, we obtained 0≤ep≤l. Nevertheless, an overlarge length may involve irrelevant signals, which lead to poor generalization ability and cause higher computing costs. In practice, the lengths of the segment and the effective period are mainly determined by engineering experience. Furthermore, in order to explore the influence of data length on the CLC method, a bias b was introduced to represent the sample length in ep, resulting in 0≤b≤ep.

**Figure 6:**
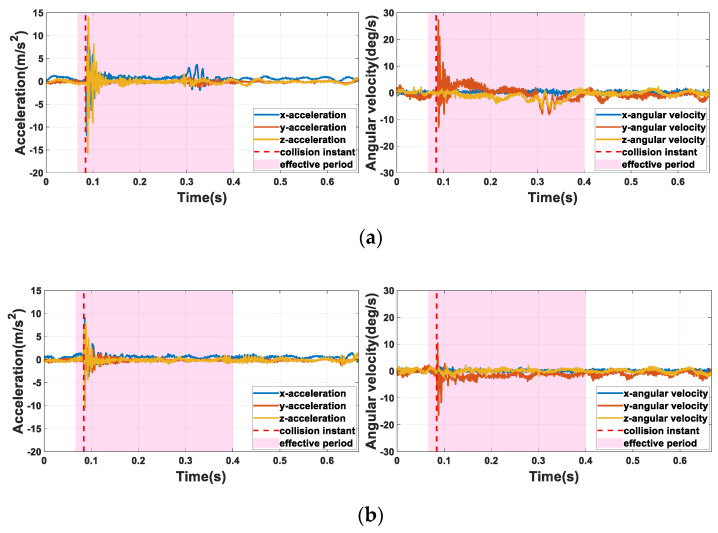

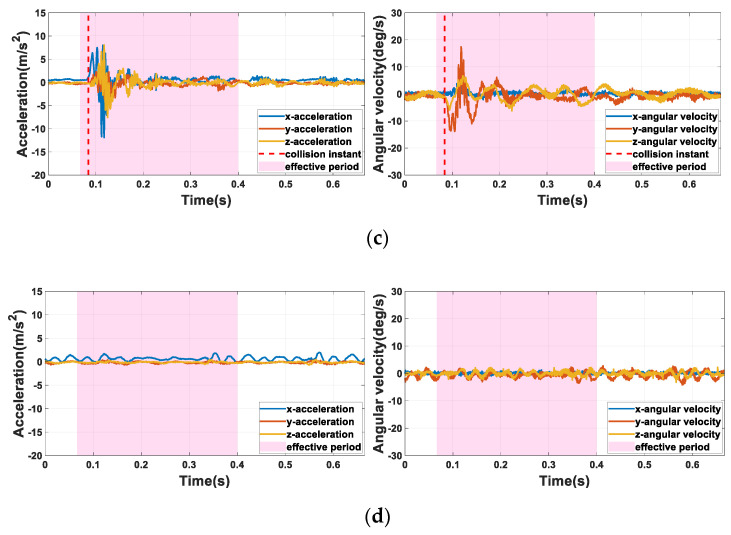
(**a**) The waveform of *vulnerable* point; (**b**) the waveform of *acceptable* point; (**c**) the waveform of *normal* point; (**d**) the waveform of *free*.

In this paper, the l of the segments was pre-determined as 666.7 ms. With the sample rate of 1500 Hz, l represents 1000 sample points. To determine the value of ep, we inspected the waveform of the pVRI signals presented in Figure 6. From Figure 6a, we can see that obvious vibration was excited by friction between the charger and the charging port 200 ms after the initial impact, especially in the vulnerable case. In order to obtain sufficient post-collision contact information, *ep* in the *vulnerable* point case was set as 333.3 ms, among which the pre-collision period was designed as approximately 20 ms so that the transient characteristics of the collisions could be captured without introducing too much irrelevant information into the system. To ensure the consistency of the length of input data, in cases of both an *acceptable* point (Figure 6b) and *normal* point (Figure 6c), the same strategy as above was used. For collision classification, as shown in Figure 6d, we also created signal segments without any collision and labeled them as *free.*

**Figure 7:**
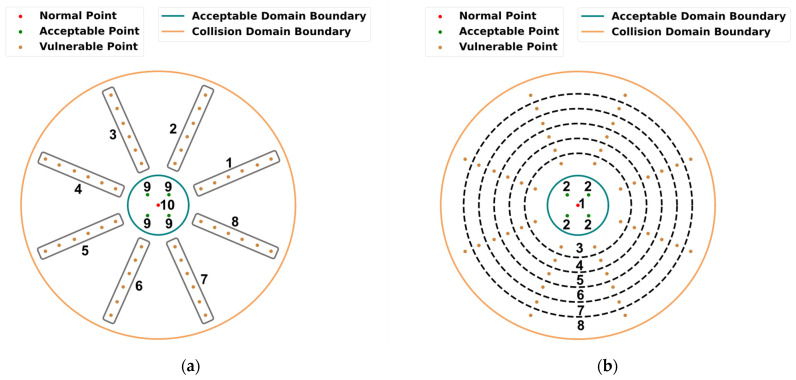
(**a**) The label diagram for collision localization along the circumferential direction; (**b**) the label diagram for collision localization along the radial direction.

*Contact* and *free* mentioned above are proposed for collision classification. Based on the different possible results after collision, *contact* can also be divided into *acceptable* contact and *vulnerable* contact. The former corresponds to the *normal* point and *acceptable* point, and the latter corresponds to the *vulnerable* point. It should be noted that in this scenario, collision classification analysis is based on the deviation, and the estimation of the deviation is based on collision localization. In other words, collision localization is the basis of collision classification here. Thus, it is necessary to label collision points in more detail for collision localization. In order to better explore the collision localization ability of the proposed method, two cases are discussed here: (1) collision localization in the circumferential direction; (2) collision localization in the radial direction. To illustrate, we define the prefix of the label in these two cases as C and R, respectively. The final label is composed of the prefix mentioned above and the number in Figure 7. According to the above definition, in Case 1, the *vulnerable* points were labeled counterclockwise as C1-C8, as shown in Figure 7a, which means that the points in the same radial direction were labeled with the same class. As for the acceptable domain, we were more concerned with the difference between the acceptable domain and the vulnerable domain, so the collision points in the acceptable domain were not labeled along the circumference. Specifically, the *normal* point was labeled as C10, and four *acceptable* points were labeled as C9. *Free* was labeled as C0 in this case. Similarly, in Case 2, *vulnerable* points were labeled as R3-R8, as shown in Figure 7b, which means that the points on the same cycle were labeled with the same class. For the same reason as in Case 2, four *acceptable* points were labeled as R2, the *normal* point was labeled as R1, and *free* was labeled as R0. At this point, two data sets based on the different annotation methods were established.

## 5. Methodology for CLC

### 5.1. Theoretical Basis

#### 5.1.1. Convolutional Neural Network

The convolutional neural network was first proposed by Le Cun in 1989 [42] and, recently, has been widely applied in many fields, such as face recognition [43], path detection [44] and fault diagnosis [45]. Moreover, due to the excellent feature extraction ability of CNN, outstanding performances have been shown in all these fields.

**Figure 8:**
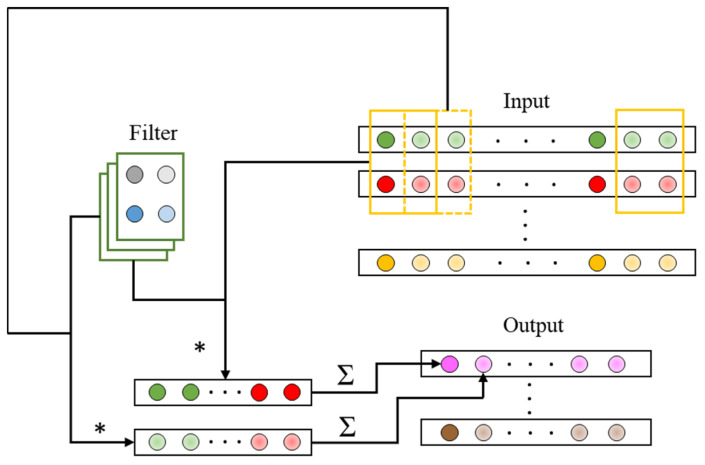
The essential components of CNN for dealing with time series, where * denotes the multiplication of elements at corresponding positions of the matrices.

The standard convolution operation on the N-dimensional time series is shown in Figure 8. Assuming that the length of the time series is $L_{s}$, the convolutional layer will take the input as a $N\times L_{s}$ feature map F. Assuming a stride of 1 and ignoring padding, the output feature map can be expressed as:

(9) $$O_{m,n}=\sum_{i,j,c}K_{i,j,c}\cdot F_{m+i-1,n+j-1}$$

where $K_{i,j,c}$, $O_{m,n}$ and $L_{k}$ denote the element in the ith row and jth column of the cth convolution filter, the element in the mth row and nth column of the output matrix and the size of the convolution filter, respectively, $m=1,\;\ldots ,\;L_{s}-L_{k}$, $n=1,\;\ldots ,\;N-L_{k}$. Note that each convolution filter is applied to all the input. The output results of each filter in the same position are superimposed together to form the new feature. All these new features are usually taken as the input of the next layer.

In general, learning results tend to become better as the depth of the network increases. However, stacking more layers usually means higher costs. In industrial applications, sometimes, a trade-off may be needed between network depth and learning effect according to the actual situation.

#### 5.1.2. Support Vector Machine

The support vector machine (SVM) was originally introduced by Vapnik [46]. It is a very powerful machine learning method that is based on the structural risk minimization principle. For two-class classification problems, it is applied to find an optimal hyperplane with the maximum margin between the support hyperplanes of different classes. As followed by the structural risk minimization principle, it can effectively reduce overfitting and improve model generalization ability [47].

For convenience, a two-class classification problem is used to illustrate the classification process. We assume the following data set:

(10) $$C=\left\{\left(x_{1},y_{1}\right),\left(x_{2},y_{2}\right),\ldots ,\left(x_{n},y_{n}\right)\;\right\}$$

where $\left(x_{i},y_{i}\right)$ is the $i_{\mathrm{th}}$ data point, $x_{i}\in R^{n}$ is the ith feature vector, which is regarded as the input of the model, and $y_{i}\in \left\{-1,+1\right\}$ is the ith class label. Assuming that the above given data set is linearly separable and addressing the corresponding QP problem by interval maximization, the separating hyperplane of the SVM can be expressed as follows:

(11) w·x+b=0

and the classification decision function can be expressed as follows:

(12) f(x)=sgn((w·x)+b)

where $w\in R^{n}$ is the vector that is normal to the separating hyperplane and b∈R is a bias term.

However, nonlinear signals are generated after robot collisions and these signals are usually linearly inseparable. To deal with the above problem, the kernel trick was introduced into the SVM. The idea of the kernel trick is to map linearly inseparable data into a new space where the transformed data can be linearly separated. Additionally, the linearly classification method could then be applied to train the data.

Moreover, to address multi-class classification problems, partitioning strategies [48] were introduced into the SVM, which includes one-verse-one (OVO), one-verse-rest (OVR) and rest-verse-one (RVO). Among these strategies, OVR is the most commonly used. For an N-class classification task, OVR converts the task into a series of binary classification tasks in which the data of any class and the remaining data of (N-1) classes are regarded as two new classes, respectively. In this way, multi-class classification problems can be handled by the SVM.

In recent years, the SVM has already achieved excellent performance in many applications, such as image classification [49], pedestrian detection [50] and pattern classification [51]. However, traditional training processes rely more on manual feature design, which requires expert experience. Moreover, using similar signals to deal with different problems often requires different artificial features. This also poses great challenges to the training process.

### 5.2. CLC Method Based on CNN and SVM

**Figure 9:**
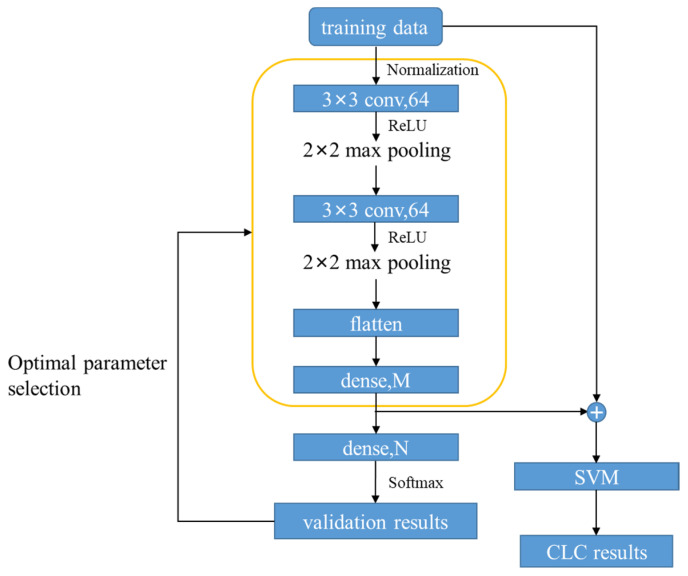
The structure of DCNN–SVM.

In this paper, we propose a CLC method composed of a double-convolutional-layer CNN and SVM (DCNN–SVM). The structure of the DCNN–SVM is shown in Figure 9. The structure was divided into two parts: a feature extractor and a classifier. In the feature extraction part, the convolutional layers had 64 3×3 filters. A stride of 1 was set for the convolutional layers. A 2×2 max pooling layer with the stride of 1 was introduced behind each convolutional layer to down-sample the input representation. Additionally, this is helpful in preventing overfitting to some degree. The activation function was applied on the output of the convolutional layers to introduce nonlinear factors into the model and improve the ability of the model to process nonlinear data. Here, the ReLU function was chosen as the activation function. The output of the final max pooling layer was flattened and then entered into the fully connected layer. The feature extraction part ended with an M-way fully connected layer and an N-way fully connected layer with softmax. The optimal parameters of the feature extractor were selected according to the prediction accuracy of the model on the validation set. In the classification part, the training data need to be extracted by the trained feature extractor, of which the parameters do not vary with training. As shown in Figure 9, the part in the yellow box is the final feature extractor. It is worth noting that the flattened data were not directly used as the extracted features as an excessive feature dimension will result in a high computation cost of the SVM. The fully connected layer was applied at the end of the extractor to reduce the feature dimension. Additionally, the extracted features were then regarded as the input to train the SVM classifier.

Since DCNN–SVM is a combination of the DCNN and SVM, the computational complexity of the DCNN and SVM should be first analyzed separately, and then the results should be combined to represent the total complexity of DCNN–SVM. The total computational complexity of all convolutional layers can be expressed as follows [52]:

(13) $$O\left(\sum_{l=1}^{d}n_{l-1}\cdot s_{l}^{2}\cdot n_{l}\cdot m_{l}^{2}\right)$$

where d is the number of convolutional layers and l is the index of a convolutional layer. $n_{l}$ is the number of filters in the lth layer, $s_{l}$ is the spatial size of the filter, and $m_{l}$ is the spatial size of the output feature map. Considering a fully connected neural network layer with I input nodes and M output nodes, the computational complexity can be described as O(IM) [53]. In terms of the SVM, given an input matrix $X\epsilon R^{c\times h}$ representing the coordinates of c points in h dimensions, the computational complexity can be expressed as $O\left(max\left(c,h\right)min{\left(c,h\right)}^{2}\right)$ [54]. Therefore, the computational complexity of DCNN–SVM can be expressed as follows:

(14) $$O\left(\sum_{l=1}^{2}n_{l-1}\cdot s_{l}^{2}\cdot n_{l}\cdot m_{l}^{2}+IM+max\left(c,h\right)min{\left(c,h\right)}^{2}\right)$$

## 6. Simulated Experiment and Results

### 6.1. Implementation

**Figure 10:**
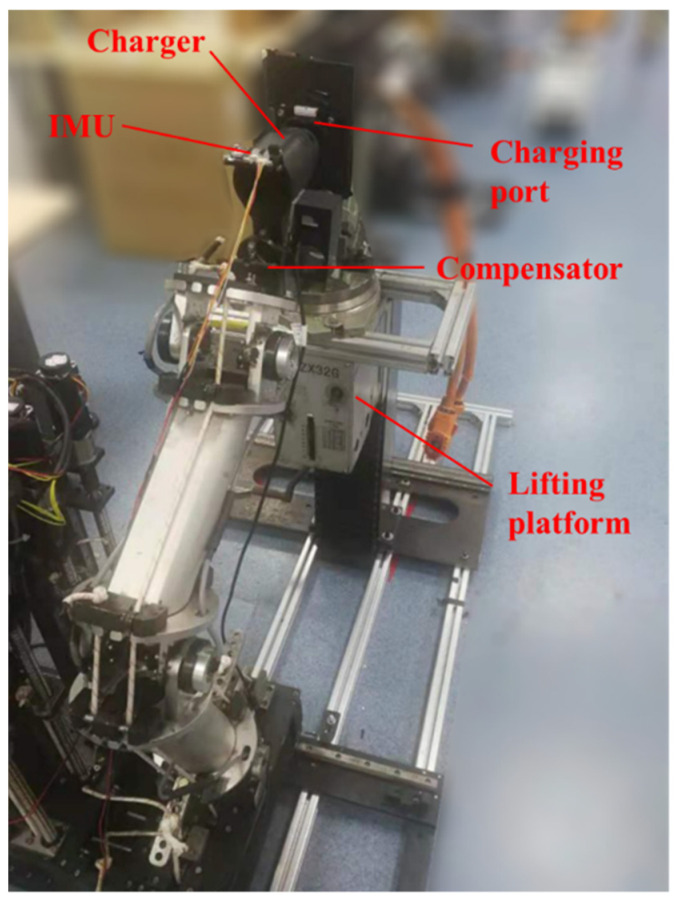
Simulated collision experiment on the serial cable-driven manipulator.

As shown in Figure 10, in the experiment, all the data were collected using the IMU mounted on the top of the charger. The charger was connected with the cable-driven manipulator using the compensator. Due to the very small deformation of the charger after collision, the collected data mainly included the vibration information from the compensator and the cable-driven manipulator. The manipulator was controlled using the PI scheme designed in [6], which includes two controllers controlling motors and cables, respectively. In the collision simulation experiment, the end effector of the manipulator moved in a straight line at a speed of 16.7 mm/s.

In terms of the quantity of data collection, in order to reduce the impact of repeated positioning errors of the manipulator on the results, in each group, each *normal* point was collected 40 times. For the same reason, each *acceptable* point and each *vulnerable* point were collected 30 times, respectively. Meanwhile, due to the obvious characteristic differences between *contact* and *free*, as shown in Figure 6, not too many *free* samples were needed. The ratio of *free* and *contact* samples was set as 1:2 in the experiment. The sample distribution in each group is listed in Table 2. To explore the influence of different joint configurations on the results, one group was randomly selected as the testing data set, which is never used in a single training process. The remaining three groups were shuffled and divided into the training data set and validation data set in a ratio of 8:2.

To illustrate the effectiveness of the proposed DCNN–SVM algorithm, we compared the results with a long short-term memory (LSTM) model [55] and the plain CNN in DCNN–SVM. These three methods were essentially automatic feature extraction methods, and the inputs were generated by normalizing the raw signals. For the DCNN–SVM, of which the structure is illustrated in Figure 9, M was set as 1024, and N was set as equal to the total number of the classes. For maxpooling layers in DCNN–SVM, the stride was set as 2 and the padding method was set as “same”. For the SVM in DCNN–SVM, by grid search, the penalty coefficient was set as 6 and the kernel was set as “rbf”. The learning rate was set as 0.0001, and the optimizer of the extractor part was set as Adam. For the CNN model design, its parameters were set as consistent with those of the DCNN–SVM. Thus, we refer to the plain CNN as DCNN here. For the LSTM model design, the structure is composed of an LSTM layer, three fully connected layers and finally a softmax layer. The LSTM layer was set as the first layer. The number of the hidden units of the LSTM layer was set as 110. The LSTM layer converted the initial input into the high-dimensional output feature matrix. The output of the LSTM layer was flattened and then fed into three fully connected layers whose sizes were 5000-way, 500-way and N-way, respectively. N was equal to the number of the classes. The learning rate was set as 0.0001, and the optimizer was set as Adam. All these three models were trained and tested using the Tensorflow 2.0 library. Other than the above two comparison models, the results of our proposed model were also compared with SVM and k-nearest neighbors (kNN) models, which are both artificial feature extraction methods. The features selected in these two models are similar to those in [17]. In more detail, the features are listed in Table 3. In addition, using the grid search method, the penalty coefficient of the SVM here was set as 10. Using the same method, for kNN, the number of neighbors was set as 7, the leaf size was set as 1 and “distance” was chosen as weights. To test these two models, we used the machine learning library from scikit-learn. To clearly describe the hyper-parameters of the mentioned methods, relative settings are listed in Table 4.

As mentioned in Section 5.2, the computational complexity of the DCNN model can be expressed as follows:

(15) $$O\left(\sum_{l=1}^{2}n_{l-1}\cdot s_{l}^{2}\cdot n_{l}\cdot m_{l}^{2}+IM\right)$$

On the other hand, the computational complexity of LSTM mainly depends on the number of weights per time step and the length of inputs. Given a number of weights w and the length of inputs i, the complexity of the LSTM layer can be expressed as O(wi). Considering the fully connected layers, the complexity of the compared LSTM model can be expressed as O(wi+IM). Furthermore, given the number of training instances g and the dimensionality of training space k, the computational complexity of kNN can be expressed as O(gk) [56].

In this experiment, we used three cross-validation to train and validate models. According to the accuracy of the validation results, we chose the best models in the DCNN–SVM, CNN and LSTM methods. For the SVM and the kNN models, we used the grid search method to obtain the optimal hyper-parameters. For each group, the process was repeated three times, and the results from each testing set were averaged. To explore the influence of b on the prediction results, we also created several test sets with various b values, which represented the segmented vibration signals with different proportions of ep. Here, we set 0.0667 s ≤b≤ 0.3333 s and the interval was set as 0.0133 s.

### 6.2. Results and Discussion

**Figure 11:**
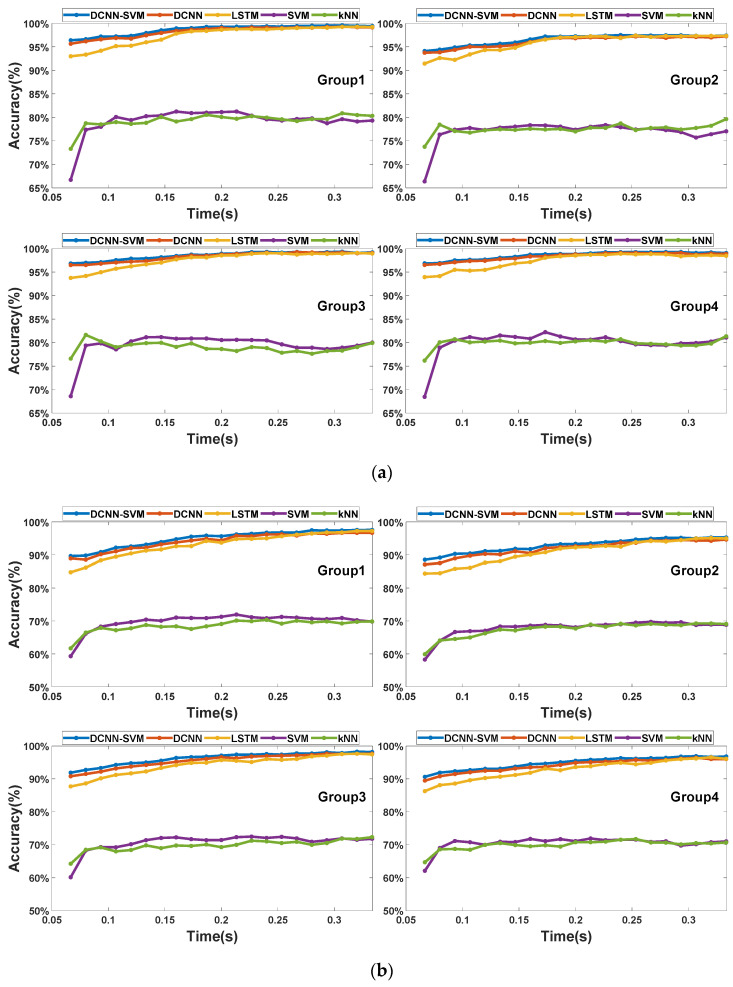
(**a**) Comparison of accuracy scores in Case 1; (**b**) comparison of accuracy scores in Case 2.

To illustrate, we define the situation shown in Figure 7a and the situation shown in Figure 7b as Case 1 and Case 2, respectively. The accuracy scores of the testing data sets with different b values are illustrated in Figure 11. In both cases, the automatic feature extraction methods performed much better than the artificial feature extraction methods, including the SVM and the kNN methods. The maximum accuracy scores of the two artificial feature extraction methods were lower than 85% in Case 1 and lower than 80% in Case 2. In contrast, the minimum accuracy scores of the three feature extraction methods were higher than 90% in Case 1 and higher than 80% in Case 2. Among the three automatic feature extraction methods, the DCNN–SVM worked best with various b values. As shown in Figure 11a, when b≤0.175 s, the accuracy scores of the three automatic feature extraction methods increased rapidly with the increase in the b value. When b>0.175 s, the accuracy scores tended to be stable. As shown in Figure 11b, when b≤0.25 s, the accuracy scores of the three automatic feature extraction methods increased rapidly with the increase in b values. When b>0.25 s, the accuracy scores tended to be stable. In both cases, in the stage of rapid growth in accuracy, the LSTM model performed poorly compared with the DCNN and the DCNN–SVM models, while, in the stage of accuracy tending to be stable, the LSTM model showed a similar effect to the DCNN and the DCNN–SVM models. To some extent, this means that the feature extractor that does not pay too much attention to time features has a better effect when collision information is insufficient. The reason for the above results is that the vulnerable and acceptable domains were divided based on spatial location. In the actual collision process, the contact mode of the two domains in the early stage of the collision was highly similar, so the collision signals of the two domains were also extremely similar in a very short period after collision, as shown in Figure 6. Therefore, it is difficult to extract sufficient effective features that can distinguish this similarity using the artificial feature extraction method, as corroborated by the results. In contrast, the features extracted by automatic feature extraction methods have higher dimensions, and therefore the possibility of extracting effective features is greater. However, it should be noted that at the early stage of collision, vibration signals have a high variation frequency, and sufficient time-related information may be not able to be obtained at the current sampling frequency. Thus, excessive attention to time-related features may make the model more sensitive to the specific features of some samples, resulting in a decrease in the generalization ability of the model. This is also related to the poorer performance of LSTM compared with the other two models when there is less collision information. Based on this point, using DCNN alone is also an alternative choice for this CLC problem.

**Figure 12:**
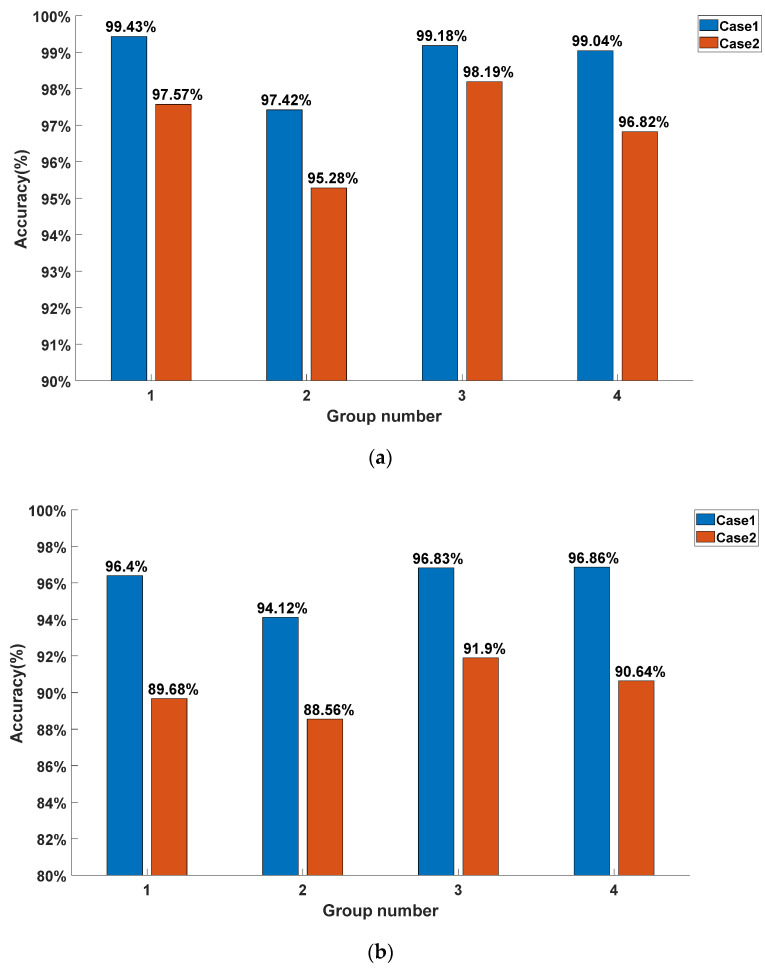
(**a**) The prediction accuracy of each group achieved by the DCNN–SVM model when b=0.3333 s; (**b**) the prediction accuracy of each group achieved by the DCNN–SVM model when b=0.0667 s.

The accuracy scores in different groups achieved by the DCNN–SVM model are shown in Figure 12. Here, we choose the situation in which b=0.3333 s and b=0.0667 s. As shown in Figure 12a,b, there were significant differences in the accuracy of different groups. This means that the joint configuration has an impact on our proposed CLC method. Furthermore, calculating the standard deviation of the accuracy scores of different groups in both cases, we could deduce that when b=0.3333 s, the standard deviations were 0.91% and 1.25%, respectively, in Cases 1 and 2, and when b=0.0667 s, the standard deviations were 1.3% and 1.42%, respectively, in Cases 1 and 2. This indicates that our method is robust, to some extent, against the influence of different joint configurations. Moreover, the smaller the b was, the smaller the standard deviation was, which reveals that sufficient collision information reduces the influence of the joint configuration on the prediction results of the DCNN–SVM model. When b was the same, the standard deviations were also different in these two cases. This means that the robustness of the DCNN–SVM model on joint configurations varies in different CLC tasks. Furthermore, we observed that the model’s performance in Case 1 was better than that in Case 2. This may be because, when the collision occurred on the collision point in the same radial direction, the direction of the resultant torque of the elastic compensator was more similar. In contrast, the collision occurred on the collision point in the same circumferential direction, but could not contribute the same property to the collision information. From the results, the situation in which the model had a better CLC effect in the circumferential direction was Case 1, which is consistent with the actual physical situation.

**Figure 13:**
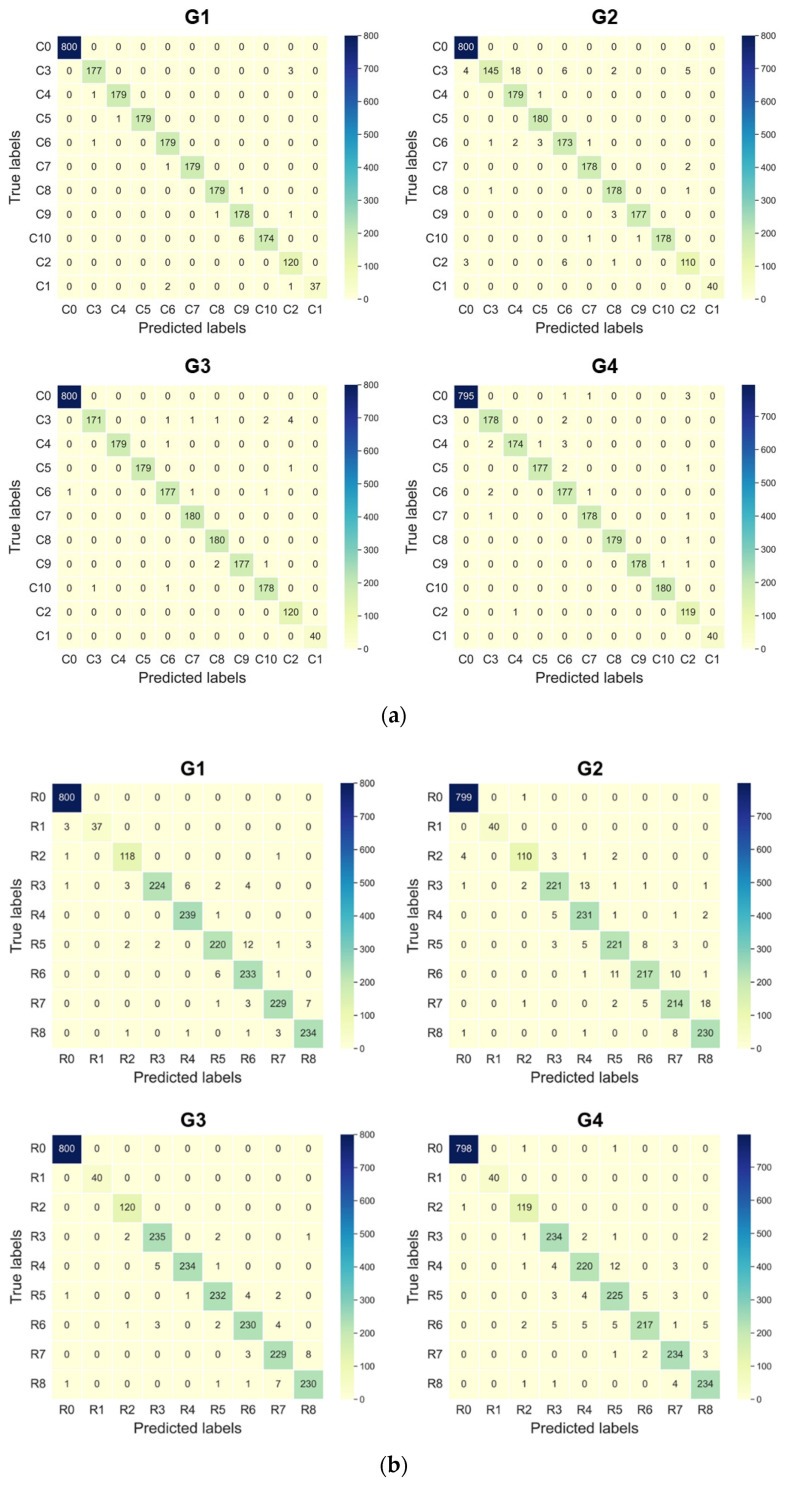
(**a**) Confusion matrices of different groups in Case 1; (**b**) confusion matrices of different groups in Case 2.

Hereinbefore, the prediction results of the collision localization and the collision classification have been discussed at the same time. Here, we conducted a more specific analysis of the collision classification and the collision localization, respectively. We chose the DCNN–SVM model with the best effect compared with other models as the analysis object. The results above were averaged, and in the process, three models needed to be trained to obtain each result. To illustrate, we chose one model from the three. The confusion matrices of the collision localization and the collision classification in Cases 1 and 2 are shown in Figure 13a,b, respectively. In Figure 13a, as illustrated in Section 4, C1 and C2 represent the situation when contact occurs in the acceptable domain, C3-C10 represent the situation when collision occurs in the vulnerable domain, and C0 represents the situation when no contact occurs. In Figure 13b, R1 and R2 represent the situation when contact occurs in the acceptable domain, R3-R8 represent the situation when collision occurs in the vulnerable domain, and R0 represents the situation when no contact occurs. The prediction precisions of the DCNN–SVM model for the three situations in both cases are listed in Table 5. The precisions of *free* and *vulnerable* were higher than 99% in both cases. Low misjudgment rates of *free* and *vulnerable* can reduce the possibility of misstopping the manipulator. In contrast, the prediction precisions of *acceptable* were lower, and the minimum precision was as low as 94.94%. From the confusion matrices in Figure 13, we can also see that the mistake mainly occurred in mispredicting some *vulnerable* instances as *acceptable* instances. The reason for this result is that our *vulnerable* and *acceptable* domains were defined based on their geometry position, and the collision modes in these two situations were much more similar at the boundary of these two domains, in contrast to *free* and *normal* instances. Thus, the likelihood of misjudgment at the boundary was greater. This can also be seen from Figure 13b, in which more R3 instances were wrongly judged as R2 instances than other instances in the rest of the vulnerable domain. This kind of mistake may cause serious damage to the manipulator. Additionally, how to improve the prediction precision of *acceptable* should be the focus of our follow-up research. In collision localization, we neglected the *free* instances. The prediction precision in Cases 1 and 2 is, respectively, listed in Table 6 and Table 7. In Case 1, the mean precision of each group was higher than or equal to 96.82%, and in Case 2, the mean precision of each group was higher than or equal to 94.12%. This means that, to some extent, our proposed method can effectively deal with the collision localization problems that occur on the end effector. Note that in Case 1, the mean precision of each group was higher than that of the same group in Case 2. This means that the DCNN–SVM model performed better in collision localization along the circumferential direction than along the radial direction. Additionally, this result is consistent with the above overall analysis of CLC.

In order to explore the influence of the kernel size of the convolutional layer on our proposed method, we selected DCNN–SVM with three different convolution kernels to conduct CLC for collision signals with b=0.0667 s and b=0.2666 s. Our experiments used Windows on the following system: Processor: Intel (R) Core (TM) i7-10700K CPU @ 3.80 GHz, Memory: 31.9 GiB, GPU: NVIDIA GeForce RTX 3080. The results in different cases are listed in Table 8 and Table 9, respectively. By comparing the accuracy of CLC with different models, we can see that the model with the 6×6 convolution kernel was slightly better than that with the 3×3 convolution kernel, while it was significantly better than the model with the 2×2 convolution kernel. In terms of run time, to predict a single sample, the time consumed by models with different convolution kernels was similar. The above results indicate that an increase in convolution kernel size is helpful to improve the performance of the model. This may be because the convolution kernel with a large size can fuse vibration signals from more dimensions together in a single sampling, which is conducive to the extraction of more effective features.

## 7. Conclusions

In this paper, we propose a model-independent CLC method for the end effectors of cable-driven manipulators, which is composed of a double-layer CNN and SVM, i.e., the DCNN–SVM method. The vibration signals of the compensator were chosen for the construction and training of the DCNN–SVM model. To collect a reasonable data set, the dynamic characteristics of the cable-driven manipulators were analyzed. To explore the influence of the labeling method on DCNN–SVM’s prediction results, two labeling schemes are proposed. The final test results of the simulation experiment show promising value in improving the safety of the cable-driven manipulators applied in the auto-charging scenario, even when the contact surface is irregular during collision. The conclusions may be summarized in more detail as follows:

1. The DCNN–SVM method, which can extract features automatically, performs better than the artificial feature extraction methods with various lengths of inputs. The more sufficient the collision information is, the better the DCNN–SVM’s performance. Meanwhile, in comparison to LSTM, we can see that at the early stage of the collision, the automatic feature extraction method, which is less sensitive to time-related features, works better on this CLC problem.
2. When joint configuration varies, the proposed CLC method is less affected. This means that using the vibration signals of the compensator to train the proposed method can isolate the influence of the joint configuration to some degree.
3. For different labeling schemes, the prediction precision of the proposed method is different. A smaller number of classes does not mean better performance regarding CLC problems. This may be related to the characteristics of the signal itself. Moreover, it means that some specific labeling scheme may further improve the prediction level.

It should be noted that the development of the CLC scheme, including data collection, labeling and model training, was conducted using a specific cable-driven manipulator with a specific compensator. Thus, the applicability of the proposed CLC method to different cable-driven manipulators with different compensators requires further investigation. Since the length of collision information contained in the inputs can affect the prediction accuracy of the collision localization and classification, an interesting topic is how to achieve better performance regarding CLC problems with less collision information. In this paper, the simulated auto-charging scene is that in which the central axis of the charger is perpendicular to the front of the charging port. However, in practice, the situation may change with the variation in the parking offset. Thus, we will collect collision data based on more experimental situations in future work.

## Figures and Tables

**Table 1:** The joint configurations of different groups.

Group Number	Joint Number	Joint Angle (°)
G1	$q_{J_{1}}$	−25.1
	$q_{J_{2}}$	27.1
	$q_{J_{3}}$	−1.9
G2	$q_{J_{1}}$	−16.9
	$q_{J_{2}}$	13
	$q_{J_{3}}$	3.8
G3	$q_{J_{1}}$	−15
	$q_{J_{2}}$	0.84
	$q_{J_{3}}$	14.1
G4	$q_{J_{1}}$	−32.6
	$q_{J_{2}}$	37.7
	$q_{J_{3}}$	−5.04

**Table 2:** The distribution of the collected data set.

Group Number	Normal	Acceptable	Vulnerable	Free
G1	40	120	1440	800
G2	40	120	1440	800
G3	40	120	1440	800
G4	40	120	1440	800

**Table 3:** Manual features for training kNN and SVM models.

Features in the Time Domain	Features in the Frequency Domain
Mean value	Mean frequency
Variance	Fundamental frequency
Skewness	Spectral amplitude corresponding to mean frequency
Kurtosis	Spectral amplitude corresponding to fundamental frequency
Standard deviation	Phase angle corresponding to mean frequency
Median value	Phase angle corresponding to fundamental frequency
Extreme range	Crest factor
Extreme deviation	Average signal angle
Energy increasing rate	-

**Table 4:** Settings of the hyper-parameters of the compared models.

Model	Hyper-Parameters	Settings
DCNN–SVM	Kernel size	3×3
	Striding and padding	2, “same”
	Learning rate	0.0001
	Mini-batch size for training	32
	Optimizer	Adam
	Loss function	Cross-entropy
	Maximum epochs	70
	Kernel of the SVM	RBF
	Penalty coefficient of the SVM	6
DCNN	Kernel size	3×3
	Striding and padding	2, “same”
	Learning rate	0.0001
	Mini-batch size for training	32
	Optimizer	Adam
	Loss function	Cross-entropy
	Maximum epochs	70
LSTM	Number of hidden units	110
	Learning rate	0.0001
	Mini-batch size for training	32
	Optimizer	Adam
	Loss function	Cross-entropy
	Maximum epochs	70
SVM	Kernel of the SVM	RBF
	Penalty coefficient of the SVM	10
KNN	Number of neighbors	7
	Leaf size	3
	Weights	“distance”

**Table 5:** Prediction precision of DCNN–SVM for collision classification.

Class	Case	Precision of G1	Precision of G2	Precision of G3	Precision of G4
Free	1	100%	100%	99.88%	100%
	2	99.38%	99.25%	99.75%	99.87%
Vulnerable	1	99.86%	99.5%	100%	99.79%
	2	99.93%	99.55%	100%	99.93%
Acceptable	1	97.53%	94.94%	96.97%	95.78%
	2	96.27%	97.4%	98.16%	96.36%

**Table 6:** Prediction precision of DCNN–SVM for collision localization in Case 1.

Class	Precision of G1	Precision of G2	Precision of G3	Precision of G4
C1	100%	100%	100%	100%
C2	96%	93.22%	96%	96.75%
C3	98.88%	98.64%	99.42%	97.27%
C4	99.44%	89.95%	100%	99.43%
C5	100%	97.83%	100%	99.44%
C6	98.35%	93.51%	98.33%	96.20%
C7	100%	98.89%	98.9%	99.44%
C8	99.44%	96.74%	98.36%	100%
C9	96.22%	99.44%	100%	100%
C10	100%	100%	97.8%	99.45%
Mean precision	98.83%	96.82%	98.88%	98.80%

**Table 7:** Prediction precision of DCNN–SVM for collision localization in Case 2.

Class	Precision of G1	Precision of G2	Precision of G3	Precision of G4
R1	100%	100%	100%	100%
R2	95.16%	97.35%	97.56%	95.97%
R3	99.12%	95.26%	96.71%	94.74%
R4	97.15%	91.66%	99.57%	95.24%
R5	95.65%	92.86%	97.48%	92.21%
R6	92.09%	93.94%	96.64%	96.88%
R7	97.45%	90.68%	94.63%	95.51%
R8	95.9%	91.27%	96.23%	95.9%
Mean precision	96.57%	94.12%	97.35%	95.81%

**Table 8:** Prediction accuracy of DCNN–SVM for CLC with different convolution kernels in Case 1.

b	Kernel Size	Acc. of G1	Acc. of G2	Acc. of G3	Acc. of G4	Mean Acc.	Num of Conv1param ^1^	Num of Conv2param ^1^	Run Time
0.0667 s	2×2	99.29%	97.29%	98.92%	99.08%	98.65%	320	16,448	2.23 ms
	3×3	99.38%	97.13%	99.33%	99.38%	98.8%	640	36,928	2.26 ms
	6×6	99.38%	97.42%	99.25%	99.29%	98.84%	2368	147,520	2.33 ms
0.2666 s	2×2	95.38%	93.25%	96.04%	96.21%	95.22%	320	16,448	1.68 ms
	3×3	95.96%	94.71%	96.75%	96.54%	95.99%	640	36,928	1.73 ms
	6×6	96.75%	94.38%	96.58%	96.83%	96.14%	2368	147,520	1.67 ms

^1^ Conv1param and Conv2param represent parameters of convolutional layers 1 and 2, respectively.

**Table 9:** Prediction accuracy of DCNN–SVM for CLC with different convolution kernels in Case 2.

b	Kernel Size	Acc. of G1	Acc. of G2	Acc. of G3	Acc. of G4	Mean Acc.	Num of Conv1param ^1^	Num of Conv2param ^1^	Run Time
0.0667 s	2×2	95.88%	92.79%	96.96%	95.46%	95.27%	320	16,448	2.42 ms
	3×3	96.5%	94.88%	97.88%	96.29%	96.39%	640	36,928	2.47 ms
	6×6	97.54%	95.33%	97.71%	96.71%	96.82%	2368	147,520	2.49 ms
0.2666 s	2×2	87.75%	86.21%	89.71%	88.71%	88.1%	320	16,448	2.09 ms
	3×3	89.71%	87.54%	91.63%	90%	89.72%	640	36,928	2.04 ms
	6×6	90.79%	88.5%	91.83%	90.88%	90.5%	2368	147,520	1.97 ms

^1^ Conv1param and Conv2param represent parameters of convolutional layers 1 and 2, respectively.

## Data Availability

The data mentioned in this paper are provided.

## References

[1] Paulius D., Sun Y. (2019). A Survey of Knowledge Representation in Service Robotics. Robot. Auton. Syst..

[2] Pinillos R., Marcos S., Feliz R., Zalama E., Gómez-García-Bermejo J. (2016). Long-Term Assessment of a Service Robot in a Hotel Environment. Robot. Auton. Syst..

[3] Sung H.J., Jeon H.M. (2020). Untact: Customer’s Acceptance Intention toward Robot Barista in Coffee Shop. Sustainability.

[4] Walzel B., Sturm C., Fabian J., Hirz M., Bargende M., Reuss H.-C., Wiedemann J. (2016). Automated Robot-Based Charging System for Electric Vehicles. Proceedings of the 16th Internationales Stuttgarter Symposium.

[5] Miseikis J., Ruther M., Walzel B., Hirz M., Brunner H. (2017). 3D Vision Guided Robotic Charging Station for Electric and Plug-In Hybrid Vehicles. arXiv.

[6] Lou Y., Di S. (2020). Design of a Cable-Driven Auto-Charging Robot for Electric Vehicles. IEEE Access.

[7] Bryson J.T. The Optimal Design of Cable-Driven Robots. 121. 29 Jan 2016. https://udspace.udel.edu/handle/19716/17757.

[8] Lou Y., Wei J., Song S. (2019). Design and Optimization of a Joint Torque Sensor for Robot Collision Detection. IEEE Sens. J..

[9] Haddadin S., De Luca A., Albu-Schaffer A. (2017). Robot Collisions: A Survey on Detection, Isolation, and Identification. IEEE Trans. Robot..

[10] Wiig M.S., Pettersen K.Y., Krogstad T.R. (2020). Collision Avoidance for Underactuated Marine Vehicles Using the Constant Avoidance Angle Algorithm. IEEE Trans. Contr. Syst. Technol..

[11] Fan T., Long P., Liu W., Pan J. (2020). Distributed Multi-Robot Collision Avoidance via Deep Reinforcement Learning for Navigation in Complex Scenarios. Int. J. Robot. Res..

[12] Johnson J.K. (2018). On the Relationship between Dynamics and Complexity in Multi-Agent Collision Avoidance. Auton. Robot..

[13] Xiao J., Zhang Q., Hong Y., Wang G., Zeng F. (2018). Collision Detection Algorithm for Collaborative Robots Considering Joint Friction. Int. J. Adv. Robot. Syst..

[14] Gordić Z., Jovanović K. (2020). Collision Detection on Industrial Robots in Repetitive Tasks Using Modified Dynamic Time Warping. Robotica.

[15] Popov D., Klimchik A., Mavridis N. (2017). Collision Detection, Localization & Classification for Industrial Robots with Joint Torque Sensors. Proceedings of the 2017 26th IEEE International Symposium on Robot and Human Interactive Communication (RO-MAN).

[16] Vorndamme J., Schappler M., Haddadin S. (2017). Collision Detection, Isolation and Identification for Humanoids. Proceedings of the 2017 IEEE International Conference on Robotics and Automation (ICRA), Marina Bay Sands.

[17] Zhang Z., Qian K., Schuller B.W., Wollherr D. (2021). An Online Robot Collision Detection and Identification Scheme by Supervised Learning and Bayesian Decision Theory. IEEE Trans. Automat. Sci. Eng..

[18] Ren T., Dong Y., Wu D., Chen K. (2018). Collision Detection and Identification for Robot Manipulators Based on Extended State Observer. Control Eng. Pract..

[19] Golz S., Osendorfer C., Haddadin S. (2015). Using Tactile Sensation for Learning Contact Knowledge: Discriminate Collision from Physical Interaction. Proceedings of the 2015 IEEE International Conference on Robotics and Automation (ICRA).

[20] De Luca A., Albu-Schaffer A., Haddadin S., Hirzinger G. (2006). Collision Detection and Safe Reaction with the DLR-III Lightweight Manipulator Arm. Proceedings of the 2006 IEEE/RSJ International Conference on Intelligent Robots and Systems.

[21] Min F., Wang G., Liu N. (2019). Collision Detection and Identification on Robot Manipulators Based on Vibration Analysis. Sensors.

[22] De Luca A., Mattone R. (2005). Sensorless Robot Collision Detection and Hybrid Force/Motion Control. Proceedings of the Proceedings of the 2005 IEEE International Conference on Robotics and Automation.

[23] Iskandar M., Eiberger O., Albu-Schaffer A., Luca A.D., Dietrich A. Collision Detection, Identification, and Localization on the DLR SARA Robot with Sensing Redundancy. Proceedings of the 2021 IEEE International Conference on Robotics and Automation (ICRA).

[24] Tang J., Zhang Y., Huang F., Li J., Chen Z., Song W., Zhu S., Gu J. (2019). Design and Kinematic Control of the Cable-Driven Hyper-Redundant Manipulator for Potential Underwater Applications. Appl. Sci..

[25] Dahiya R.S., Mittendorfer P., Valle M., Cheng G., Lumelsky V.J. (2013). Directions toward Effective Utilization of Tactile Skin: A Review. IEEE Sens. J..

[26] De Maria G., Natale C., Pirozzi S. (2012). Force/Tactile Sensor for Robotic Applications. Sens. Actuators A Phys..

[27] Narukawa K., Yoshiike T., Tanaka K., Kuroda M. (2017). Real-Time Collision Detection Based on One Class SVM for Safe Movement of Humanoid Robot. Proceedings of the 2017 IEEE-RAS 17th International Conference on Humanoid Robotics (Humanoids).

[28] Dimeas F., Avendaño-Valencia L.D., Aspragathos N. (2015). Human–Robot Collision Detection and Identification Based on Fuzzy and Time Series Modelling. Robotica.

[29] Wolpert D.H., Macready W.G. (1997). No Free Lunch Theorems for Optimization. IEEE Trans. Evol. Comput..

[30] Cho C.-N., Kim J.-H., Kim Y.-L., Song J.-B., Kyung J.-H. (2012). Collision Detection Algorithm to Distinguish between Intended Contact and Unexpected Collision. Adv. Robot..

[31] Petmezas G., Haris K., Stefanopoulos L., Kilintzis V., Tzavelis A., Rogers J.A., Katsaggelos A.K., Maglaveras N. (2021). Automated Atrial Fibrillation Detection Using a Hybrid CNN-LSTM Network on Imbalanced ECG Datasets. Biomed. Signal Process. Control.

[32] Bahrami M., Forouzanfar M. (2022). Sleep Apnea Detection from Single-Lead ECG: A Comprehensive Analysis of Machine Learning and Deep Learning Algorithms. IEEE Trans. Instrum. Meas..

[33] Rai H.M., Chatterjee K. (2022). Hybrid CNN-LSTM Deep Learning Model and Ensemble Technique for Automatic Detection of Myocardial Infarction Using Big ECG Data. Appl. Intell..

[34] Singh K., Malhotra J. (2022). Smart Neurocare Approach for Detection of Epileptic Seizures Using Deep Learning Based Temporal Analysis of EEG Patterns. Multimed. Tools Appl..

[35] Rodriguez Aguiñaga A., Muñoz Delgado L., López-López V.R., Calvillo Téllez A. (2022). EEG-Based Emotion Recognition Using Deep Learning and M3GP. Appl. Sci..

[36] Gao S., Shi S., Zhang Y. (2022). Rolling Bearing Compound Fault Diagnosis Based on Parameter Optimization MCKD and Convolutional Neural Network. IEEE Trans. Instrum. Meas..

[37] Zhang K., Fan C., Zhang X., Shi H., Li S. (2022). A Hybrid Deep-Learning Model for Fault Diagnosis of Rolling Bearings in Strong Noise Environments. Meas. Sci. Technol..

[38] Shi Z., Hao H., Zhao M., Feng Y., He L., Wang Y., Suzuki K. (2019). A Deep CNN Based Transfer Learning Method for False Positive Reduction. Multimed. Tools Appl..

[39] Deepak S., Ameer P.M. (2019). Brain Tumor Classification Using Deep CNN Features via Transfer Learning. Comput. Biol. Med..

[40] (2017). Interoperability Test Specifications of Electric Vehicle Conductive Charging—Part 1: Supply.

[41] Rognant M., Courteille E., Maurine P. (2010). A Systematic Procedure for the Elastodynamic Modeling and Identification of Robot Manipulators. IEEE Trans. Robot..

[42] LeCun Y., Boser B.E., Denker J.S., Henderson D., Howard R.E., Hubbard W.E., Jackel L.D. Handwritten Digit Recognition with a Back-Propagation Network. Proceedings of the Advances in Neural Information Processing Systems(NIPS 1989).

[43] Ferrari C., Lisanti G., Berretti S., Del Bimbo A. (2018). Investigating Nuisances in DCNN-Based Face Recognition. IEEE Trans. Image Process..

[44] Kim W.-S., Lee D.-H., Kim Y.-J., Kim T., Hwang R.-Y., Lee H.-J. (2020). Path Detection for Autonomous Traveling in Orchards Using Patch-Based CNN. Comput. Electron. Agric..

[45] Wang H., Xu J., Yan R., Gao R.X. (2020). A New Intelligent Bearing Fault Diagnosis Method Using SDP Representation and SE-CNN. IEEE Trans. Instrum. Meas..

[46] Cortes C., Vapnik V. (1995). Support-Vector Networks. Mach. Learn..

[47] Tang L., Tian Y., Pardalos P.M. (2019). A Novel Perspective on Multiclass Classification: Regular Simplex Support Vector Machine. Inf. Sci..

[48] Tomar D., Agarwal S. (2015). A Comparison on Multi-Class Classification Methods Based on Least Squares Twin Support Vector Machine. Knowl. Based Syst..

[49] Mori K., Matsugu M., Suzuki T. Face Recognition Using SVM Fed with Intermediate Output of CNN for Face Detection. Proceedings of the MVA APR Conference on Machine Vision Applications.

[50] Szarvas M., Yoshizawa A., Yamamoto M., Ogata J. (2005). Pedestrian Detection with Convolutional Neural Networks. Proceedings of the IEEE Proceedings. Intelligent Vehicles Symposium.

[51] Zhang X., Ding S., Xue Y. (2017). An Improved Multiple Birth Support Vector Machine for Pattern Classification. Neurocomputing.

[52] He K., Sun J. Convolutional Neural Networks at Constrained Time Cost. Proceedings of the IEEE Conference on Computer Vision and Pattern Recognition (CVPR).

[53] Cheng Y., Yu F.X., Feris R.S., Kumar S., Choudhary A., Chang S.-F. (2015). An Exploration of Parameter Redundancy in Deep Networks with Circulant Projections. Proceedings of the 2015 IEEE International Conference on Computer Vision (ICCV).

[54] Chapelle O. (2007). Training a Support Vector Machine in the Primal. Neural Comput..

[55] Park K., Choi Y., Choi W.J., Ryu H.-Y., Kim H. (2020). LSTM-Based Battery Remaining Useful Life Prediction with Multi-Channel Charging Profiles. IEEE Access.

[56] Trisal S.K., Kaul A. (2019). K-RCC: A Novel Approach to Reduce the Computational Complexity of KNN Algorithm for Detecting Human Behavior on Social Networks. J. Intell. Fuzzy Syst..

